# The need for thorough phase II studies in medicines development for Alzheimer’s disease

**DOI:** 10.1186/s13195-015-0153-y

**Published:** 2015-10-26

**Authors:** Julian A. Gray, David Fleet, Bengt Winblad

**Affiliations:** Neuroglobe Ltd, Baarerstrasse 135, 6301 Zug Switzerland; Datamagik Ltd, Laburnum House, East Grimstead, Salisbury, Wiltshire SP5 3RT UK; Karolinska Institutet, Department NVS, Center for Alzheimer Research, Division for Neurogeriatrics, Novum plan 5, 14157 Huddinge, Sweden

## Abstract

An important factor in the universal failure in phase III trials in mild to moderate Alzheimer’s disease in the past decade is the lack of phase II clinical data prior to entering phase III, with common reliance on biomarker results alone. Conduction of two learn-confirm cycles according to the Sheiner model would allow go/no-go decision making to include reliable clinical efficacy data prior to conducting phase III and would likely bring the rate of late stage failure more into line with that of other neurological indications. In studies in earlier disease stages, combined phase IIB/III adaptive approaches merit consideration in view of the long timelines of each study, though advantages and disadvantages of this approach versus the classical development pathway still need careful assessment.

## Introduction

Not a single compound has successfully completed phase III in Alzheimer’s disease (AD) over the past 11 years [1, 2]. This success rate in phase III is far worse than that for the average of all neurological conditions combined, estimated to be approximately 50 % in a recent review [3]. Apart from the general difficulty of translating preclinical findings to clinical results in the AD area, there may be many reasons behind this extraordinarily high failure rate, including design issues such as potential under- or over-dosing [4, 5] and ‘regression to the mean’ after proceeding to phase III on the basis of random high phase II results [6]. More importantly, it seems that there has been a recent trend to enter phase III without adequate phase II data on clinical efficacy [2], the decision being based instead on positive effects on biomarkers.

**Fig. 1:**
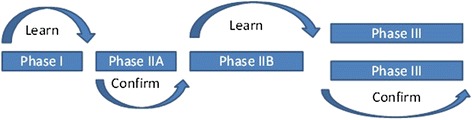
Learn-confirm model of medicines development [7, 18]. Figure reproduced with permission from [18]

A now classical model of development of new medicines (see above) entails two ‘learn-confirm’ cycles [7], as shown in Fig. 1.

According to this model, in the first cycle, initial learning occurs in phase I tests of safety and pharmacodynamics and pharmacokinetics. This is followed by confirmation of the safety and pharmacodynamics/target engagement as well as, if feasible, initial evidence of clinical efficacy in phase IIA studies in relatively small groups of patients. Information about clinical effect size and dose response is subsequently obtained in the phase IIB ‘learn phase’ of the second learn-confirm cycle, with final confirmation of efficacy and safety in larger numbers of patients in phase III studies. Inevitably the model is oversimplified, with important learning occurring throughout development. However, the basic concept remains valuable.

The duration of this full two-cycle approach may appear daunting in the case of disease-modifying studies, and efforts are often made to short-cut this by combining phases. A combined phase I/IIA study in patients could, for example, bring sufficient data on safety, pharmacokinetics and pharmacodynamics. Nevertheless, this is not sufficient as a database to commence phase III as preliminary information about clinical effect size and dose–response in a phase IIB study should first be collected to enable proper planning of these definitive studies.

Few recent AD programs have followed this model, with sponsors tending to commence a full phase III program after seeing evidence of target engagement biochemically in smaller phase II studies. The imperfect link between results on biomarkers and clinical effects observed to date makes this a high risk approach, which, if it continues to be followed by phase III failure, may lead to reduced attractiveness of the area as one for investment in the future.

In contrast, the two-cycle model was followed for currently approved agents for treating AD. For example, donepezil showed clinical efficacy in phase II [8] before conduction of phase III studies [9, 10], which were also positive.

Schneider et al. [2] have pointed out that, to date, no anti-amyloid phase III study has been preceded by a positive phase II proof of concept study. Instead the decision to move to phase III has at times been made based on positive data on effects on biomarkers rather than clinical endpoints: an example here is that of solanezumab [2], where after entering phase III studies based on biomarker and safety data in phase II, the first phase III results did not reach statistical significance (though the study led to design of the further ongoing phase III study in mild patients). Moving to phase III based on positive clinical results in subgroups but not the overall analysis in phase II also carries significant risk as exemplified by the failure in phase III of tarenflurbil in mild AD, a subgroup with apparently positive results in phase II [11]. The motivation to skip phase IIB studies prior to entry into phase III for the newer disease-modifying agents may be prompted partly by the long duration of such studies and the drive for commercial organisations to enter the market as early as possible. Another factor behind the leap into phase III may be concern over issues around statistical power in phase II. Of course, the sample size estimation is dependent on both the magnitude of the response and associated variability - which may be conveniently combined into an ‘effect size’ consisting of the mean difference between active and control groups divided by the standard deviation of the difference. Continuous development failures in the AD field have led to more cautious anticipated effect sizes - with consequently larger predicted sample sizes needed to show them.

Effect sizes of 0.4 or even less are now commonly predicted in trials of new agents for treating AD, even though larger effect sizes (0.6 or more) are commonly required in other therapeutic areas. The high variability in rates of decline on placebo between studies [12] adds to the tendency to be conservative in sample size estimation.

Concretely, in a mild to moderately demented population, use of a reliable cognitive instrument to demonstrate a modest effect size of 0.4 would require at least 100 subjects per group (two group *t*-test with 80 % power and 0.05 significance level (two tailed)). Adding to this number additional patients to compensate for a reasonably likely dropout rate (20 %), and assuming multiple dose groups (low, medium, high) with a placebo control comparator, results in a phase IIB study with approximately 500 subjects.

For agents with smaller effect sizes such studies will inevitably be underpowered to show significance at the 5 % level. However, simply demonstrating statistically significant *P* values is of lesser importance in this learning phase of development: instead, the goal should be to quantify the magnitude of treatment effect on clinical endpoints and to estimate the precision of the estimate (particularly with respect to the expected minimum effect size from the lower confidence boundary) not only across a range of endpoints and instruments but also across the different dose groups and times of assessment. Dose-dependency and consistency of effects at different time-points and across different scales and subgroups can provide support for the existence of a real drug effect.

Recent results with monoclonal antibodies directed against beta-amyloid have indicated that efficacy may be greater when such agents are applied early in the disease process. In particular, encouraging results on clinical endpoints have been obtained especially in mild rather than moderate AD patients with solanezumab in phase III studies [13], with the same trends to better response in milder patients observed in a dose-dependent manner in a 433-patient phase IIB study of crenezumab [14].

With smaller placebo declines in mild AD and in even earlier disease stages such as prodromal and preclinical AD, low power in phase II studies may become an increasingly critical issue for modestly effective compounds. Regulatory aspects of developing drugs at these early disease stages have been discussed in a recent US Food and Drug Administration guidance document [15]. Development in early AD may require new approaches. For example, involvement of patients in larger phase I studies may allow initial learning about clinical efficacy as in the recently reported positive preliminary results with aducanumab [16]. Such studies may effectively then become phase I/IIA studies as they take on some of the character of phase II studies. Likewise, it is possible to try to combine learning with confirming by using adaptive design or Bayesian approaches in a phase IIB study (which may then become a phase IIB/III study) [17] to allow discontinuation of inactive doses and adjust the sample size to ensure adequately powered final investigations whilst at the same time controlling the overall experiment-wise type I error rate. Such a study could potentially then act as a pivotal study - though at the expense of greatly increased complexity and higher potential cost associated with the larger number of patients which may eventually be included. A positive outcome at interim analysis could also trigger the start of a second phase III study. While intellectually appealing, the protracted timelines of such studies in disease-modifying trials, the inability to disclose and publicise phase II findings as well as the potential issues associated with introduction of bias at the time of interim analysis means that such approaches must be used only after careful assessment of the risks and benefits as well as discussions with regulatory authorities.

## Conclusion

During the last 10 to 15 years of clinical trials in AD we have seen an increasing tendency to enter phase III without conducting or carefully analysing adequately sized phase II studies. We recommend that phase II studies should be designed and conducted with both clinical endpoints to estimate clinical effect size, and biomarkers to establish target engagement prior to starting phase III in mild or moderately demented patients. In studies in earlier disease stages, combined phase IIB/III adaptive approaches merit consideration in view of the long timelines of each study, though advantages and disadvantages of this approach versus the classical development pathway still need careful assessment. Only by ‘looking before leaping’, can we bring down the rate of phase III failures, which have become an all too common phenomenon in medicines development for AD.

## Abbreviation

AD: Alzheimer’s disease
